# Characterizing postural oscillation in children and adolescents with hereditary sensorimotor neuropathy

**DOI:** 10.1371/journal.pone.0204949

**Published:** 2018-10-10

**Authors:** Cyntia Rogean de Jesus Alves de Baptista, Adriana Nascimento-Elias, Tenysson Will Lemos, Beatriz Garcia, Paula Domingues Calori, Ana Claudia Mattiello-Sverzut

**Affiliations:** 1 Graduate program of Rehabilitation and Functional Performance, Ribeirão Preto Medical School, University of São Paulo, Ribeirão Preto, São Paulo, Brazil; 2 Department of Health Sciences Ribeirão Preto Medical School, University of São Paulo, Ribeirão Preto, São Paulo, Brazil; 3 Physical Therapy Undergraduate Course, Ribeirão Preto Medical School, University of São Paulo, Ribeirão Preto, São Paulo, Brazil; IRCCS E. Medea, ITALY

## Abstract

Charcot Marie Tooth disease (CMT) has negative functional impact on postural control of children; however, it has not been widely studied. Stabilometry can provide insights about postural control and guide preventive interventions in immature perceptual and musculoskeletal systems as those seen in children with CMT. This cross-sectional study aimed to identify and interpret stabilometric variables that reflect the postural control of children with CMT. 53 subjects (age 6–17) were assigned to one of the two groups: CMT (15 males and 14 females with CMT) or Control (13 males and 11 females healthy). Quiet standing was tested in different conditions: with open and closed eyes on regular surface (open-regular, closed-regular) and foam surface (open-foam, closed-foam) using a force platform. The minimum of 2 and maximum of 3 trials of 30 seconds for each test condition provided the classical stabilometric variables and Romberg Quotient (RQ_v_). CMT group showed increase of confidence ellipse area, mean velocity, mediolateral and anteroposterior velocities associated with decreased mean body oscillation frequency, as the complexity of tasks increased. CMT postural deficit was identified by greater and faster sway associated with these lower frequencies, when compared to Control.

## Introduction

Adequate static and dynamic balance depend on the normal mechanisms of postural control, biomechanical factors[1] and neuromuscular factors, including the integration of visual sensory, vestibular and somatosensory information[2]. From childhood to adulthood, postural control improves with internal and /or external destabilizing forces[3–9]. The critical period for the development of postural control seems to be between 7 and 11 years of age[4], although studies in the literature have described that an adult-like postural control is reached only after the age of 14 [10–12]

An objective method of analyzing postural control is stabilometry, however the diverse number of variables and methods used by authors prevent interpretations and comparisons between studies[13–16] The position and displacement of the center of pressure (CoP), i.e the point of the reaction force when it is applied to the ground on the support base, allows for obtaining derivative variables that signal greater or lesser postural stability. For healthy children, a review based on 14 studies showed decreased postural sway with increasing age and increased sway, with the absence of visual feedback[17]. When compared to adults, children show increase in postural sway velocity and variance[18,19]. However, reference values for stabilometric parameter are not available for most ages. Maturation of bipodal postural control is expressed as a decrease in CoP amplitude oscillation[4], area of oscillation and the velocity of CoP with increasing age[4,17,20]. Studies using semi-tandem posture show that postural stability is achieved between 7 and 10 years of age and remains stable from 10 to 11 years. After that, it was considered compatible with adults[8].

Pathological conditions in children, especially neuromuscular diseases, such as neuropathies show increased anteroposterior CoP oscillation, while Duchenne muscular dystrophy shows increased mediolateral oscillation[21]. Hereditary sensory-motor neuropathies (HSMN), as Charcot-Marie-Tooth (CMT), affect 9.4 to 20 in 100 000 people [22] independently of gender. There are reports of greater postural instability on standing position, when compared to their healthy peers, manifested by greater area of CoP oscillation in adults with different polyneuropathies[23] and higher CoP velocity in the CMT1A population[24], regardless of the visual information. Van der Linden et al.[25] also found an increase in the velocity of CoP displacement indicating postural instability in adults with CMT1A, however this impairment was less pronounced, when compared to patients with diabetic neuropathy or progressive spinal amyotrophic.

Stabilometry can detect subjects with and without balance; however, the literature lacks stabilometric data in HSMN children and adolescents. This gap in the literature limits the design of important interventions, such as the development of preventive measures for distal deformities [26] and falls[27]. To date, there are no curative approaches for CMT. Physical therapy is an alternative that delays functional losses. Approaches such as, balance exercises[28,29], strength training [30,31] aerobic training[32,33] and orthosis use[34–36] are documented for the late stages of the disease. We believe that most of those approaches, including balance exercises, could be more effective, when used during childhood.

However, features of postural oscillation in children with CMT must be described for deficits that might improve with physical therapy, to be detected and adaptation mechanisms to be inferred. From the functional point of view, postural control is a prerequisite for most human daily activities. If the most common CMT types manifest during childhood, and balance may be impaired, there will be an increased propensity to comorbidities that lead to a sedentary lifestyle after sprains[31,37], falls and fractures[38–40] Thus, the present study aimed to identify and interpret stabilometric variables that assess the static postural control of children and adolescents with HSMN.

## Materials and methods

This cross-sectional study was composed of 53 children and adolescents from 6 to 17 years of age. Of the 53, 29 had hereditary sensory-motor neuropathy (HNSM), specifically Charcot Marie Tooth (CMT group) and 24 were healthy (Control group) (Table 1). Inclusion criteria for the CMT group were: a) medical diagnosis, b) independent standing c) independent gait. Inclusion criteria for the Control group was age and gender match with CMT. Exclusion criteria for the CMT group were: a) diagnosis under investigation and b) presence of comorbidities such as diabetes mellitus and hypothyroidism. Exclusion criteria for Control were: a) athletes b) presence of balance disorders, neurological or psychiatric pathology. Exclusion criteria for both groups were: a) previous orthopedic surgeries in the lower limbs, b) cognitive inability to understand and perform the tests and c) presence of respiratory diseases. Participants with vision impairment corrected by glasses were not excluded.

**Table 1:** Anthropometric features of CMT group and Control group with results of differences between groups.

	CMT group n = 29		Control Group n = 24		
	Mean (SD)	CI 95%	Mean (SD)	CI 95%	p-value
**Weight (kg)**	48.16 (16.01)	6.84	47.46 (15.25)	6.10	0.45
**Height (cm)**	150.31(13.70)	5.50	152.33(15.59)	6.24	0.39

SD = standard deviation; CI = Confidence interval

The CMT group was composed by patients from ANGE—HCFMRP (Ambulatório de Neurogenética do Hospital das Clínicas de Ribeirão Preto). The Control group subjects were recruited from local primary schools, through a written invitation and a questionnaire about health conditions. The parents or legal guardians of the children and adolescents agreed to their participation by signing a free informed consent form. The study was approved by the Ethics Committee of Hospital das Clínicas da Faculdade de Medicina de Ribeirão Preto, Universidade de São Paulo (process number 14904/2014).

We collected anthropometric data (weight and height) and used static balance tests.

Weight of participants was obtained using a digital scale (Welmy W300) and height was measured using a conventional stadiometer.

Balance tests used a force platform (Bertec—model 4060-08, Bertec Corporation—USA) to record the center of pressure (CoP) displacement at a sampling frequency of 100 Hz. The test was performed at a natural upright position (barefoot, arms along the body, natural parallel position of feet) in four different test conditions, which were randomly assigned. Each test condition comprised of three trials of 30 seconds[41]: open eyes on regular surface (open-regular); closed eyes on regular surface (closed-regular); open eyes on foam surface (open-foam); closed eyes on foam surface (closed-foam). Tests were performed in a quiet environment and recordings started when participant reached a stable standing position. Participants were instructed to maintain their natural upright position until the examiner signaled the end of the test. They made 30-second pauses between trials. For open-eyes conditions, they were requested to gaze at a target (a black circle, 5 cm in diameter) 2 meters straight ahead from their support base[42,43]. For closed-eyes condition, participants were requested to stay with their eyes closed until instructed otherwise by the examiner. The same support base was provided for all test conditions, with the use of plantar impression over an E.A.V carpet. An ordinary foam block (40x40x10 cm, density of 33 kg/m^2^) was used on open-foam and closed-foam trials.

Raw data for CoP and stabilometric parameters were calculated as described by Duarte[44], using Matlab (13.0) software. The stabilometric analysis included the following spatial series: 95% confidence ellipse area, mean velocity of the center of pressure displacement, mediolateral and anteroposterior velocity and temporal series: mean frequency, mediolateral and anteroposterior frequencies. Additionally, the mean CoP displacement velocity was used to calculate the Romberg Quotient (RQ_v)_, which estimates the contribution of vision to the postural stability on regular (closed-regular/open-regular x 100) and foam (closed-foam/open-foam x 100) surfaces. Due to its good reliability and reproducibility, when compared to other stabilometric measures [45–47], COP velocity was used to obtain the RQ.

The CoP velocity was normalized according to participant’s height[48] The confidence ellipse area and frequencies were not normalized, since it was minimally influenced by anthropometric data[42].

We used only valid data, i.e., trials of 30 seconds for each condition in which participants remained in the position stipulated for the test: relaxed and without making voluntary body movements (quiet standing). Data analysis were performed using SPSS (version 17.0) considering the mean of at least 2 trials per condition and adopting 5% level of significance. Shapiro Wilk test was used to verify the normality of the samples and Student T test was used to verify anthropometric differences between groups. Stabilometric variables were analyzed using Friedman test for intragroup comparison, followed by post hoc Wilcoxon signed rank test, with adjusted p-value corrected by Bonferroni (p = 0.008). Mann-Whitney U- test was used for intergroup comparison (CMT versus Control).

## Results

Table 1 shows Anthropometric data of participants and no statistical differences between groups.

Stabilometric parameters were evaluated considering intragroup (conditions of test within each group) and intergroup (CMT versus Control) analyses.

### Intragroup analysis

#### CMT group

Not all participants could perform all test conditions or maintain the 30 seconds of quiet standing needed for recording. We had 26 participants for open-foam and 23 for closed-foam conditions.

CMT showed lower values of confidence ellipse area for the following: open-regular, when compared to open and closed-foam, closed-regular when compared to closed-foam and open-foam (X^2^ (3) = 38.13 p<0.008). There were no statistically significant differences in confidence ellipse, when comparing open-regular and closed-regular (Table 2).

**Table 2:** Descriptive measures of stabilometric variables of CMT group.

CMT group ◊				
Variable	Condition	Median	25 ^th^ %	75 ^th^ %
**Confidence ellipse area (mm**^**2**^**)**	open-regular	626.870	184.940	949.130
	closed-regular	647.078	283.960	861.304
	open-foam	1194.034	861.250	1732.904
	closed-foam	2963.662	1684.941	4660.876
**Normalized total velocity**	open-regular	0.004	0.003	0.005
	closed-regular	0.005	0.004	0.007
	open-foam	0.006	0.004	0.006
	closed-foam	0.010	0.007	0.013
**Normalized mediolateral velocity**	open-regular	0.003	0.002	0.004
	closed-regular	0.003	0.026	0.005
	open-foam	0.004	0.003	0.005
	closed-foam	0.007	0.005	0.009
**Normalized anteroposterior velocity**	open-regular	0.002	0.001	0.003
	closed-regular	0.003	0.002	0.003
	open-foam	0.003	0.003	0.005
	closed-foam	0.006	0.004	0.008
**Total frequency (Hz)**	open-regular	0.492	0.357	0.537
	closed-regular	0.503	0.389	0.554
	open-foam	0.433	0.333	0.564
	closed-foam	0.398	0.245	0.528
**Mediolateral frequency (Hz)**	open-regular	0.792	0.503	0.858
	closed-regular	0.797	0.622	0.875
	open-foam	0.792	0.578	0.839
	closed-foam	0.785	0.495	0.809
**Anteroposterior frequency (Hz)**	open-regular	0.779	0.579	0.846
	closed-regular	0.797	0.604	0.819
	open-foam	0.795	0.483	0.868
	closed-foam	0.779	0.431	0.836

The mean velocity and mediolateral velocity increased as the complexity of the task increased, higher values were seen for: closed-foam when compared to open-foam, open-foam when compared to closed-regular and closed-regular when compared to open-regular (X^2^ (3) = 40.06 p<0.008) (Table 2). Anteroposterior velocity was higher for closed-foam, when compared to open-foam, open-foam when compared to closed-regular and open-regular. There were no statistically significant differences for open-regular, when compared to closed-regular (Table 2).

CMT did not show any significant differences for mean frequency, mediolateral and anteroposterior frequencies in the different test conditions (Table 2).

#### Control group

All participants of this group were able to perform all test conditions. The Control group showed confidence ellipse area values significantly lower for the following: open-regular, when compared to open-foam and closed-foam, closed-regular, when compared to closed-foam and closed-regular, when compared to open-foam (X^2^ (3) = 65.6, p<0.008, post hoc Wilcoxon corrected by Bonferroni, adjusted p = 0.008) (Table 3).

**Table 3:** Descriptive measures of stabilometric variables of Control group.

Control group				
Variable	Condition	Median	25 ^th^ %	75 ^th^ %
**Confidence ellipse area (mm**^**2**^**)**	open-regular	173.244	98.863	320.463
	closed-regular	213.227	142.081	365.485
	open-foam	584.758	426.557	1001.000
	closed-foam	1447.446	1069.575	2159.138
**Normalized total velocity**	open-regular	0.003	0.002	0.003
	closed-regular	0.003	0.002	0.004
	open-foam	0.004	0.003	0.005
	closed-foam	0.007	0.006	0.008
**Normalized mediolateral velocity**	open-regular	0.002	0.001	0.002
	closed-regular	0.002	0.002	0.003
	open-foam	0.003	0.002	0.003
	closed-foam	0.005	0.004	0.005
**Normalized anteroposterior velocity**	open-regular	0.001	0.001	0.017
	closed-regular	0.001	0.001	0.002
	open-foam	0.002	0.002	0.003
	closed-foam	0.005	0.004	0.005
**Total frequency (Hz)**	open-regular	0.840	0.460	0.565
	closed-regular	0.829	0.494	0.562
	open-foam	0.830	0.404	0.563
	closed-foam	0.827	0.409	0.531
**Mediolateral frequency (Hz)**	open-regular	0.524	0.770	0.859
	closed-regular	0.530	0.800	0.873
	open-foam	0.509	0.793	0.878
	closed-foam	0.480	0.788	0.858
**Anteroposterior frequency (Hz)**	open-regular	0.816	0.780	0.862
	closed-regular	0.821	0.785	0.868
	open-foam	0.834	0.795	0.872
	closed-foam	0.805	0.762	0.848

There were no significant differences for confidence ellipse area, when comparing open-regular and closed-regular for this group (Table 3). Mean velocity increased with the complexity of the task and higher values were seen for closed-foam, when compared to open-foam and open-foam, when compared to closed-regular (X^2^ (3) = 65.15 1). There were no significant differences between open-regular and closed-regular for this group (Table 3).

Mediolateral velocity increased with the complexity of the task in all the conditions and higher values were seen for: closed-foam, when compared to open-foam, open-regular and closed-regular; open-foam, when compared to closed-regular and open-regular and open-regular, when compared to closed-regular (X^2^ (3) = 65.50 p<0.008) (Table 3).

Anteroposterior velocity showed a significant increase according to the complexity of the task with higher values for closed-foam, when compared to open-foam and open-foam, when compared to closed-regular and open-regular (X^2^ (3) = 66.60 p<0.008). There was no significant difference between open-regular and closed-regular for this group (Table 3).

There was a significant difference in mean velocity between the test conditions for the Control group (X^2^ (3) = 9.55 p = 0.023). The post-hoc analysis showed higher mean frequency for open-regular, when compared to the open-foam and closed-foam conditions (Table 3). The mediolateral and anteroposterior frequencies did not show significant differences between the test conditions (Table 3).

#### Intergroup analysis

**Fig 1:**
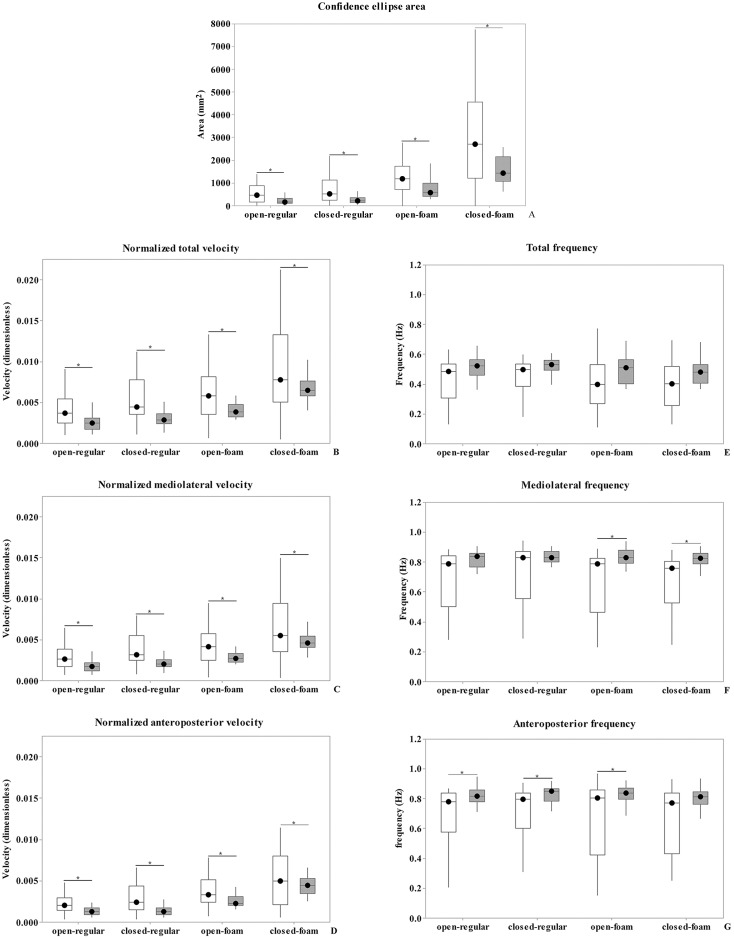
CMT and Control group comparisons of stabilometric variables at all conditions.

U-Mann Whitney showed higher values for confidence ellipse area, mean velocity, mediolateral and anteroposterior velocity for the CMT group in all test conditions, when compared to the Control group (Fig 1A–1D).

The white columns represent CMT group and the grey columns represent the Control group. Brackets were used to link conditions that presented significant differences (* p<0.08).

There were no differences between groups for total mean frequency (Fig 1E). Mediolateral frequency values showed significant difference only for open- foam (U = 188, p = 0.007) and closed-foam condition (U = 153, p = 0.003), in which CMT group showed decreased values, when compared to Control (Fig 1F). CMT group showed lower anteroposterior frequency, when compared to Control in all test conditions (open-regular—U = 218, p = 0.03; closed-regular—U = 217, p = 0.03; open-foam—U = 216, p = 0.03), except for closed-foam condition (Fig 1G).

#### Romberg quotient

Romberg Quotient (QR_v_) obtained from CoP oscillation velocity on regular (closed-regular/open-regular) and foam (closed-foam/open-foam) surfaces were not different between groups (Table 4).

**Table 4:** Romberg Quotient (QR_v_) of CMT group and Control group.

	CMT (n = 23 ^‡^)		Control (n = 24)		
RQv	Median	Minimum—Maximum	Median	Minimum—Maximum	p-value
**closed-regular / open-regular**	1,37	0,79–3,22	1,26	0,79–1,63	0,07
**closed-foam / open-foam**^‡^	0,61	0,39–2,68	0,61	0,41–1,00	0,49

^‡^ For QR_v_ based on closed-foam/open-foam, six participants were excluded of analysis because they did not achieve the minimum time to data collection.

## Discussion

The present study aimed to identify and interpret stabilometric variables that assess the static postural control of children and adolescents with CMT and shows ways of using CoP to maintain postural stability, in the presence of distal weakness and somatosensory impairments. Studies in the literature have shown that stabilometric assessment is capable of distinguishing poor and appropriate balance in children[9,49–52] and adults[23–25,53,54]. To the extent of our knowledge, this is the first original study focused on classical stabilometric parameters, in children and adolescents with CMT. Kaya et al [52]explored only the percentage of oscillation of CoP in children with Duchenne muscular dystrophy and different polyneuropathic diseases, while others have studied balance impairment in adults with neuropathies[23–25].

Since postural control is in stage of development in children, we tried to find cues about how CMT children manage the upright position, while dealing with the disadvantages that the disease imposes.

Our results show that CMT have an increase in confidence ellipse area and CoP velocity associated with the decrease of frequency in specific sensory/biomechanical conditions. There is need for more studies to expand this finding.

### Confidence ellipse area and velocity

Impaired static postural control can be inferred by confidence ellipse area and mean velocity, which was significantly greater and faster in the CMT group, when compared to the Control group, for all test conditions. Tozza et al [24]did not find increased sway area but found increased velocity of CoP in adults with CMT. Aside from the differences in methodological approach to treat CoP data[9,13–15,55–57] theses divergent findings can be due to the fact that strategies to control CoP might be different in adults, when compared to children. This might be due to different stages of CMT or postural control due to maturation. As far as the stage of the disease, it can be assumed that the joint, muscle and somatosensory deficits are more pronounced in adults, when compared to children. Unlike adults, children have preserved plantar flexion strength and distal range of movement. A previous study from our laboratory with CMT children found correlation between balance and joint/ muscle deficits[58,59] It is possible that children have increased confidence ellipse area and velocity at the expense of residual triceps surae activity, something that could be investigated by correlating clinical, kinematic and electromyographic data in future studies. Healthy adults control stance by increasing the muscular activity of the triceps surae, when under sensory deprivation on the soles of the feet[60] which could be correlated to children, at the early stages of neuropathic diseases. There are many unanswered questions about how this population manages sensory-motor deficits inherent to the polyneuropathy in postural development. However, in general, the greater the area and the greater the velocity, the more the postural instability [10,14,18,24,47] and this suggests that our neuropathic group (CMT) seems to have difficulties with postural control. Stabilometric parameters and intragroup differences help clarify this idea. CMT children showed higher mean velocity in tests using closed eyes, when compared to tests using open eyes. Curiously, our RQ_v_ data did not confirm the differences between CMT and their controls. This finding corroborates the findings of Tozza et al (2016)[24], that did not find differences in RQ of CMT and healthy adults.

CMT showed increased mediolateral velocity according to task complexity, a pattern that was similar in our healthy children, corroborating other developmental studies of postural control[17,20]. Anteroposterior velocity was also increased, with the exception of the open-regular and closed -regular conditions. Also, it is important to note that some children with CMT could not perform tasks with their eyes closed, on foam or both (closed-foam condition) suggesting limitation to maintain balance. This was not the case for the control group in which 100% of participants completed all tests. The difference in COP velocity between CMT and controls, due to the different sensorial test conditions, suggests that this is not a problem with postural control development, but differences in the availability of sensory information available for these two groups. This hypothesis is partially supported by van der Linden et al (2010)[25] who compared adult subjects with different types of postural instability, such as motor (spinal Muscular Atrophy) and sensorimotor deficiency (CMT), and Lencioni et al (2015)[54] who studied CMT. In addition, foot type in children may modify the availability of somatosensory information[61] and this is currently under investigation in our research group.

It is well-known that sensory conditions affect COP velocities in adults, more pronounced way in the anteroposterior, when compared to the mediolateral direction[53,62] Our study showed velocities being affected in both directions for children with CMT, evidenced by a higher magnitude of responses, when compared to their controls. For conditions with foam pads, we expected to see a significant reduction in the variability of velocities and area, as seen in other studies in the literature[18,63]. However, such decrease of variability was not observed in the CMT group, instead the magnitude of variables and its variability increased for conditions with foam pads, suggesting impaired capacity to control posture under somatosensory input constraints.

### Frequencies

When the frequency of CoP oscillation is analyzed as a whole, children with CMT do not differ from Control.

However, considering the direction of CoP oscillation, mediolateral frequency was lower in all conditions for CMT, when compared to Control. The same happened with anteroposterior frequency, except for closed-foam, suggesting that the constraint imposed by the disease could not be compensated in this last condition. From the sensory processing standpoint, this corroborates findings in the literature which show that only the vestibular input is available in closed-foam conditions, which has limited contribution to quiet standing[8].

The CoP frequency reflects the body mass vibration and anteroposterior frequency and is related to ankle control, which is increased in healthy children, when compared to healthy adults[64] Mediolateral frequency is related to the loading and unloading mechanism of hip control[65] While the clinical meaning of frequency remains unclear, it seems that the higher the frequencies the better the postural control. Reduced mediolateral frequency has been found in children with Duchenne muscular dystrophy[52]. In the present study mediolateral frequency on regular surface, in children with CMT, is similar to healthy children and it might be explained by CMT characteristics (preserved proximal function—hip muscle strength). On foam surface, CMT showed lower mediolateral frequency, when compared to the Control group, suggesting the use of increased stiffness strategy[66,67] to deal with the demand of the task. Moreover, reduced anteroposterior frequency suggests poor management of the upright position by the ankle joint. The biomechanical demands that foam surfaces put on the subjects[68,69], the loss of ankle passive and active range of motion observed in HNSM, could partly explain this finding[58].

One of the limitations of this study was the low number of participants analyzed at more challenging test conditions such as, closed-foam and open- foam (n = 23), because children with CMT did not achieve the 30 seconds needed. Also, our study did not investigate the spectral frequency bands. This type of analysis could reveal the preponderance of a specific sensory system in postural control, as shown in other pathological conditions[56,57,70]. Another limitation for studies with CMT subjects, including this one, is the heterogeneity of CMT impairments and different levels of sensory-motor maturation in children. The last issue was attenuated by the gender-age pairing between the CMT and the healthy children.

## Conclusion

The study shows that low postural control in children with hereditary sensory motor neuropathy can be identified by greater and faster sway, when compared to their controls. Children with CMT choose to reduce the frequency of body oscillation to deal with their standing position, especially when the sensory references are restricted.

## Supporting information

S1 File Confidence ellipse area, COP velocities, mean frequencies and Romberg quotient of each participant and condition. (DOCX)
