# Supplementary material for: Characterizing postural oscillation in children and adolescents with hereditary sensorimotor neuropathy
Source: PLoS One. 2018 Oct 10;13(10):e0204949. doi: 10.1371/journal.pone.0204949 (PMC6179247; doi:10.1371/journal.pone.0204949)
Supplement: S1 File — (DOCX) [file pone.0204949.s001.docx]

**S1 Table 1. Confidence ellipse area of each participant and condition.**

Legend: CEA=confidence ellipse area; NCEA = normalized confidence ellipse area; OR= open regular condition; CR= closed -regular condition; OF= open-foam condition; CF = closed-foam condition. Empty cels are missing data.

| **Subject** | **GROUP** | **AGE** | **CEA OR** | **NCEA OR** | **CEA CR** | **NCEA CR** | **CEA OF** | **NCEA OF** | **CEA CF** | **NCEA CF** |
| --- | --- | --- | --- | --- | --- | --- | --- | --- | --- | --- |
| 1 | CMT | 7 | 1385.97738 | 0.00083 | 861.30347 | 0.00052 | 865.11760 | 0.00052 | 2401.96657 | 0.00144 |
| 2 | CMT | 7 | 408.04763 | 0.00028 | 209.45269 | 0.00014 | 8.00273 | 0.00001 | 9.06053 | 0.00001 |
| 3 | CMT | 7 | 966.87536 | 0.00050 | 687.61096 | 0.00036 | 3832.05632 | 0.00198 | 3917.48223 | 0.00203 |
| 4 | CMT | 8 | 319.44686 | 0.00017 | 261.63034 | 0.00014 | 856.42105 | 0.00046 | 2238.92288 | 0.00119 |
| 5 | CMT | 9 | 184.94072 | 0.00009 | 283.96118 | 0.00014 | 861.25729 | 0.00042 | 990.21597 | 0.00048 |
| 6 | CMT | 9 | 474.68313 | 0.00022 | 488.60945 | 0.00023 | 1187.91162 | 0.00056 | 2963.66145 | 0.00139 |
| 7 | CMT | 9 | 812.43526 | 0.00047 | 1388.53304 | 0.00080 | 1883.09803 | 0.00108 | 4660.87616 | 0.00267 |
| 8 | CMT | 10 | 949.13226 | 0.00045 | 2182.13376 | 0.00104 | 2137.08703 | 0.00102 | 5752.75876 | 0.00274 |
| 9 | CMT | 10 | 355.42988 | 0.00015 | 821.70816 | 0.00035 | 5020.87154 | 0.00212 | 5817.88991 | 0.00245 |
| 10 | CMT | 10 | 734.71074 | 0.00029 | 3661.38506 | 0.00147 | 2772.08159 | 0.00111 |  |  |
| 11 | CMT | 10 | 458.63169 | 0.00020 | 387.50812 | 0.00017 | 988.15457 | 0.00044 | 2829.08252 | 0.00126 |
| 12 | CMT | 10 | 3494.37249 | 0.00224 | 22414.58263 | 0.01435 | 3912.60274 | 0.00250 |  |  |
| 13 | CMT | 11 | 71.16051 | 0.00004 | 364.44642 | 0.00019 | 574.44657 | 0.00029 | 1684.94119 | 0.00086 |
| 14 | CMT | 11 | 318.68663 | 0.00012 | 536.10703 | 0.00020 | 1190.59059 | 0.00045 | 3033.23327 | 0.00116 |
| 15 | CMT | 12 | 102.88801 | 0.00005 | 128.62320 | 0.00006 | 441.89264 | 0.00020 | 740.37065 | 0.00034 |
| 16 | CMT | 14 | 640.72958 | 0.00028 | 780.88273 | 0.00035 | 1111.95541 | 0.00049 |  |  |
| 17 | CMT | 14 | 829.53419 | 0.00034 | 791.01663 | 0.00033 | 1418.85404 | 0.00058 | 2557.28087 | 0.00105 |
| 18 | CMT | 14 | 75.60704 | 0.00003 | 64.21229 | 0.00003 | 13.48454 | 0.00001 | 2.78785 | 0.00000 |
| 19 | CMT | 14 | 297.95424 | 0.00011 | 462.00585 | 0.00018 | 1732.90418 | 0.00066 | 3890.58260 | 0.00148 |
| 20 | CMT | 15 | 1011.89903 | 0.00040 | 3342.40200 | 0.00131 | 971.01992 | 0.00038 | 1311.32436 | 0.00051 |
| 21 | CMT | 15 | 14.34644 | 0.00001 | 18.67793 | 0.00001 | 30.94696 | 0.00001 | 30.76338 | 0.00001 |
| 22 | CMT | 16 | 712.48877 | 0.00023 | 1431.17337 | 0.00047 | 1552.57814 | 0.00051 | 5855.66385 | 0.00191 |
| 23 | CMT | 16 | 1316.75733 | 0.00047 | 3778.05103 | 0.00134 | 1541.13009 | 0.00055 | 4523.49426 | 0.00160 |
| 24 | CMT | 17 | 47.93795 | 0.00002 | 36.33129 | 0.00001 | 495.66910 | 0.00019 | 1550.54803 | 0.00059 |
| 25 | CMT | 15 | 162.02145 | 0.00005 | 58.23324 | 0.00002 | 63.07556 | 0.00002 | 40.20373 | 0.00001 |
| 26 | CMT | 11 | 167.16701 | 0.00006 | 202.83739 | 0.00007 | 947.26801 | 0.00035 | 2597.81199 | 0.00095 |
| 27 | CMT | 10 | 951.00466 | 0.00048 | 336.49412 | 0.00017 | 1197.47828 | 0.00060 | 3258.18522 | 0.00164 |
| 28 | CMT | 14 | 626.86972 | 0.00026 | 656.27384 | 0.00027 | 1757.46660 | 0.00073 | 7761.70664 | 0.00323 |
| 29 | CMT | 15 | 635.00210 | 0.00025 | 647.07751 | 0.00026 | 1527.45575 | 0.00061 | 9933.47618 | 0.00398 |
| 30 | Control | 7 | 322.82340 | 0.00018 | 302.77687 | 0.00017 | 1509.55660 | 0.00084 | 2568.27959 | 0.00143 |
| 31 | Control | 7 | 563.43188 | 0.00035 | 640.29312 | 0.00040 | 1309.03539 | 0.00082 | 1749.33653 | 0.00110 |
| 32 | Control | 7 | 202.14385 | 0.00012 | 549.77096 | 0.00034 | 1139.91426 | 0.00070 | 1433.66246 | 0.00088 |
| 33 | Control | 9 | 240.55492 | 0.00013 | 321.56304 | 0.00018 | 490.85536 | 0.00027 | 643.51829 | 0.00036 |
| 34 | Control | 9 | 312.20392 | 0.00017 | 527.25801 | 0.00029 | 1088.03061 | 0.00059 | 2326.62550 | 0.00126 |
| 35 | Control | 9 | 141.59526 | 0.00006 | 140.92955 | 0.00006 | 892.43196 | 0.00038 | 3911.32197 | 0.00167 |
| 36 | Control | 9 | 96.71523 | 0.00005 | 145.53536 | 0.00008 | 577.73257 | 0.00030 | 756.22198 | 0.00039 |
| 37 | Control | 10 | 328.66557 | 0.00015 | 380.12558 | 0.00017 | 845.74251 | 0.00038 | 1461.22962 | 0.00066 |
| 38 | Control | 11 | 142.19273 | 0.00007 | 264.21453 | 0.00013 | 554.57945 | 0.00026 | 1166.26794 | 0.00055 |
| 39 | Control | 10 | 313.38221 | 0.00013 | 511.49233 | 0.00022 | 1037.18496 | 0.00044 | 2374.08785 | 0.00101 |
| 40 | Control | 10 | 585.63941 | 0.00027 | 499.22697 | 0.00023 | 1854.59344 | 0.00085 | 4592.74494 | 0.00210 |
| 41 | Control | 9 | 90.76915 | 0.00005 | 165.24857 | 0.00008 | 439.79622 | 0.00022 | 986.16649 | 0.00050 |
| 42 | Control | 10 | 307.42375 | 0.00015 | 297.69776 | 0.00015 | 639.04912 | 0.00032 | 1409.21095 | 0.00071 |
| 43 | Control | 12 | 144.34371 | 0.00005 | 210.29810 | 0.00007 | 422.51955 | 0.00014 | 1847.67920 | 0.00062 |
| 44 | Control | 12 | 105.30502 | 0.00004 | 97.32918 | 0.00004 | 522.79349 | 0.00021 | 770.08045 | 0.00032 |
| 45 | Control | 14 | 244.46059 | 0.00008 | 288.73455 | 0.00010 | 809.81117 | 0.00028 | 2228.58842 | 0.00077 |
| 46 | Control | 14 | 138.45646 | 0.00005 | 79.87218 | 0.00003 | 438.66718 | 0.00016 | 1950.78653 | 0.00070 |
| 47 | Control | 14 | 86.15136 | 0.00003 | 162.59584 | 0.00006 | 295.25263 | 0.00010 | 1026.18708 | 0.00036 |
| 48 | Control | 13 | 55.88925 | 0.00002 | 58.62708 | 0.00002 | 348.72796 | 0.00014 | 1068.37189 | 0.00043 |
| 49 | Control | 15 | 77.26194 | 0.00003 | 92.22910 | 0.00004 | 334.03001 | 0.00013 | 1393.80789 | 0.00055 |
| 50 | Control | 16 | 537.41545 | 0.00020 | 216.15606 | 0.00008 | 614.50722 | 0.00023 | 1474.35760 | 0.00055 |
| 51 | Control | 16 | 36.30621 | 0.00001 | 54.90095 | 0.00002 | 345.85220 | 0.00010 | 1073.18530 | 0.00032 |
| 52 | Control | 17 | 517.72000 | 0.00018 | 173.95221 | 0.00006 | 591.78389 | 0.00021 | 1647.72110 | 0.00058 |
| 53 | Control | 17 | 122.53977 | 0.00005 | 172.59682 | 0.00007 | 334.62226 | 0.00013 | 1211.88725 | 0.00047 |

**S1 Table 2. COP velocities of each participant and condition.**

Legend: VML=mediolateral velocity; NVML = normalized mediolateral velocity; VAP= anteroposterior velocity; NVAP= normalized anteroposterior velocity; VT= total velocity; NVT= normalized total velocity; OR= open regular condition; CR= closed -regular condition; OF= open-foam condition; CF = closed-foam condition. Empty cels are missing data.

| **Subject** | **GROUP** | **AGE** | **VML OR** | **NVML OR** | **VML CR** | **NVML CR** | **VML OF** | **NVML OF** | **VML CF** | **NVML CF** | **VAP OR** | **NVAP OR** | **VAP CR** | **NVAP CR** | **VAP OF** | **NVAP OF** | **VAP CF** | **NVAP CF** | **VT OR** | **NVT OR** | **VT CR** | **NVT CR** | **VT OF** | **NVT OF** | **VT CF** | **NVT CF** |
| --- | --- | --- | --- | --- | --- | --- | --- | --- | --- | --- | --- | --- | --- | --- | --- | --- | --- | --- | --- | --- | --- | --- | --- | --- | --- | --- |
| 1 | CMT | 7 | 14.651 | 0.004 | 15.745 | 0.004 | 15.453 | 0.004 | 31.411 | 0.009 | 19.619 | 0.006 | 17.838 | 0.005 | 19.637 | 0.006 | 28.051 | 0.008 | 20.720 | 0.006 | 22.266 | 0.006 | 21.854 | 0.006 | 44.422 | 0.012 |
| 2 | CMT | 7 | 13.391 | 0.004 | 10.052 | 0.003 | 2.589 | 0.001 | 2.727 | 0.001 | 44.727 | 0.013 | 31.746 | 0.009 | 4.328 | 0.001 | 4.474 | 0.001 | 18.938 | 0.005 | 14.216 | 0.004 | 3.661 | 0.001 | 3.857 | 0.001 |
| 3 | CMT | 7 | 11.296 | 0.003 | 12.454 | 0.003 | 20.045 | 0.005 | 33.060 | 0.009 | 10.335 | 0.003 | 8.293 | 0.002 | 28.664 | 0.008 | 29.620 | 0.008 | 15.975 | 0.004 | 17.613 | 0.005 | 28.347 | 0.008 | 46.753 | 0.013 |
| 4 | CMT | 8 | 5.315 | 0.001 | 6.769 | 0.002 | 8.887 | 0.002 | 14.454 | 0.004 | 5.407 | 0.001 | 4.867 | 0.001 | 8.723 | 0.002 | 15.278 | 0.004 | 7.517 | 0.002 | 9.573 | 0.003 | 12.568 | 0.003 | 20.441 | 0.006 |
| 5 | CMT | 9 | 6.208 | 0.002 | 10.246 | 0.003 | 16.807 | 0.004 | 20.382 | 0.005 | 4.384 | 0.001 | 5.671 | 0.002 | 13.564 | 0.004 | 13.365 | 0.004 | 8.780 | 0.002 | 14.490 | 0.004 | 23.769 | 0.006 | 28.824 | 0.008 |
| 6 | CMT | 9 | 13.635 | 0.004 | 18.747 | 0.005 | 16.148 | 0.004 | 35.167 | 0.009 | 9.189 | 0.002 | 8.940 | 0.002 | 12.602 | 0.003 | 23.384 | 0.006 | 19.283 | 0.005 | 26.512 | 0.007 | 22.837 | 0.006 | 49.734 | 0.013 |
| 7 | CMT | 9 | 13.588 | 0.004 | 20.549 | 0.006 | 21.220 | 0.006 | 39.729 | 0.011 | 9.024 | 0.003 | 10.879 | 0.003 | 17.988 | 0.005 | 34.974 | 0.010 | 19.216 | 0.005 | 29.061 | 0.008 | 30.009 | 0.008 | 56.185 | 0.016 |
| 8 | CMT | 10 | 24.149 | 0.006 | 48.494 | 0.013 | 28.372 | 0.008 | 56.504 | 0.015 | 17.878 | 0.005 | 22.313 | 0.006 | 19.686 | 0.005 | 31.797 | 0.008 | 34.152 | 0.009 | 68.581 | 0.018 | 40.124 | 0.011 | 79.909 | 0.021 |
| 9 | CMT | 10 | 8.013 | 0.002 | 15.401 | 0.004 | 33.045 | 0.009 | 39.048 | 0.010 | 8.379 | 0.002 | 13.988 | 0.004 | 47.798 | 0.012 | 44.350 | 0.011 | 11.332 | 0.003 | 21.780 | 0.006 | 46.733 | 0.012 | 55.222 | 0.014 |
| 10 | CMT | 10 | 16.096 | 0.004 | 31.073 | 0.008 | 24.279 | 0.006 | #DIV/0! | #DIV/0! | 9.791 | 0.002 | 18.026 | 0.005 | 21.165 | 0.005 | #DIV/0! | #DIV/0! | 22.763 | 0.006 | 43.943 | 0.011 | 34.335 | 0.009 | #DIV/0! | #DIV/0! |
| 11 | CMT | 10 | 8.695 | 0.002 | 10.673 | 0.003 | 14.378 | 0.004 | 27.367 | 0.007 | 7.052 | 0.002 | 5.930 | 0.002 | 10.684 | 0.003 | 21.417 | 0.006 | 12.296 | 0.003 | 15.093 | 0.004 | 20.333 | 0.005 | 38.702 | 0.010 |
| 12 | CMT | 10 | 25.922 | 0.007 | 53.589 | 0.015 | 32.954 | 0.009 | #DIV/0! | #DIV/0! | 25.745 | 0.007 | 151.660 | 0.043 | 44.764 | 0.013 | #DIV/0! | #DIV/0! | 36.659 | 0.010 | 75.787 | 0.022 | 46.604 | 0.013 | #DIV/0! | #DIV/0! |
| 13 | CMT | 11 | 3.827 | 0.001 | 6.287 | 0.002 | 11.170 | 0.003 | 20.329 | 0.005 | 3.098 | 0.001 | 4.422 | 0.001 | 10.736 | 0.003 | 16.489 | 0.004 | 5.412 | 0.001 | 8.892 | 0.002 | 15.797 | 0.004 | 28.750 | 0.008 |
| 14 | CMT | 11 | 7.491 | 0.002 | 11.566 | 0.003 | 14.411 | 0.004 | 29.385 | 0.007 | 5.964 | 0.001 | 8.879 | 0.002 | 13.073 | 0.003 | 29.067 | 0.007 | 10.595 | 0.003 | 16.357 | 0.004 | 20.381 | 0.005 | 41.557 | 0.010 |
| 15 | CMT | 12 | 8.038 | 0.002 | 9.996 | 0.003 | 8.036 | 0.002 | 13.327 | 0.004 | 3.615 | 0.001 | 4.501 | 0.001 | 5.295 | 0.001 | 7.046 | 0.002 | 11.368 | 0.003 | 14.137 | 0.004 | 11.365 | 0.003 | 18.848 | 0.005 |
| 16 | CMT | 14 | 13.962 | 0.004 | 24.877 | 0.006 | 19.493 | 0.005 | #DIV/0! | #DIV/0! | 7.810 | 0.002 | 9.605 | 0.003 | 15.114 | 0.004 | #DIV/0! | #DIV/0! | 19.745 | 0.005 | 35.182 | 0.009 | 27.567 | 0.007 | #DIV/0! | #DIV/0! |
| 17 | CMT | 14 | 10.519 | 0.003 | 9.963 | 0.003 | 15.674 | 0.004 | 18.618 | 0.005 | 8.202 | 0.002 | 9.095 | 0.002 | 12.580 | 0.003 | 19.417 | 0.005 | 14.876 | 0.004 | 14.090 | 0.004 | 22.167 | 0.006 | 26.330 | 0.007 |
| 18 | CMT | 14 | 8.018 | 0.002 | 7.910 | 0.002 | 1.741 | 0.000 | 1.501 | 0.000 | 16.427 | 0.004 | 14.308 | 0.004 | 2.968 | 0.001 | 2.365 | 0.001 | 11.340 | 0.003 | 11.186 | 0.003 | 2.463 | 0.001 | 2.122 | 0.001 |
| 19 | CMT | 14 | 6.934 | 0.002 | 9.816 | 0.002 | 13.670 | 0.003 | 19.589 | 0.005 | 4.992 | 0.001 | 5.430 | 0.001 | 9.912 | 0.002 | 16.685 | 0.004 | 9.806 | 0.002 | 13.882 | 0.003 | 19.333 | 0.005 | 27.703 | 0.007 |
| 20 | CMT | 15 | 10.700 | 0.003 | 19.008 | 0.005 | 10.648 | 0.003 | 14.238 | 0.004 | 6.205 | 0.002 | 10.303 | 0.003 | 7.993 | 0.002 | 8.474 | 0.002 | 15.131 | 0.004 | 26.881 | 0.007 | 15.058 | 0.004 | 20.136 | 0.005 |
| 21 | CMT | 15 | 4.308 | 0.001 | 4.226 | 0.001 | 7.192 | 0.002 | 6.476 | 0.002 | 5.757 | 0.001 | 5.969 | 0.002 | 10.200 | 0.003 | 7.152 | 0.002 | 6.092 | 0.002 | 5.976 | 0.002 | 10.171 | 0.003 | 9.158 | 0.002 |
| 22 | CMT | 16 | 10.482 | 0.003 | 20.151 | 0.005 | 18.654 | 0.005 | 39.488 | 0.010 | 6.405 | 0.002 | 11.614 | 0.003 | 17.394 | 0.004 | 36.181 | 0.009 | 14.824 | 0.004 | 28.497 | 0.007 | 26.380 | 0.006 | 55.844 | 0.013 |
| 23 | CMT | 16 | 19.700 | 0.005 | 63.351 | 0.016 | 23.734 | 0.006 | 60.318 | 0.015 | 12.270 | 0.003 | 26.631 | 0.007 | 14.078 | 0.003 | 32.453 | 0.008 | 27.860 | 0.007 | 89.592 | 0.022 | 33.564 | 0.008 | 85.303 | 0.021 |
| 24 | CMT | 17 | 2.928 | 0.001 | 3.229 | 0.001 | 8.483 | 0.002 | 12.364 | 0.003 | 1.619 | 0.000 | 1.538 | 0.000 | 4.978 | 0.001 | 8.273 | 0.002 | 4.141 | 0.001 | 4.567 | 0.001 | 11.996 | 0.003 | 17.485 | 0.004 |
| 25 | CMT | 15 | 11.934 | 0.003 | 13.948 | 0.003 | 8.924 | 0.002 | 9.411 | 0.002 | 16.847 | 0.004 | 15.359 | 0.004 | 11.634 | 0.003 | 11.553 | 0.003 | 16.878 | 0.004 | 19.726 | 0.005 | 12.620 | 0.003 | 13.309 | 0.003 |
| 26 | CMT | 11 | 4.148 | 0.001 | 7.309 | 0.002 | 12.209 | 0.003 | 24.016 | 0.006 | 5.316 | 0.001 | 6.926 | 0.002 | 13.092 | 0.003 | 24.798 | 0.006 | 5.867 | 0.001 | 10.337 | 0.003 | 17.266 | 0.004 | 33.964 | 0.008 |
| 27 | CMT | 10 | 14.084 | 0.004 | 11.137 | 0.003 | 14.752 | 0.004 | 26.775 | 0.007 | 10.674 | 0.003 | 7.139 | 0.002 | 14.494 | 0.004 | 23.574 | 0.006 | 19.918 | 0.005 | 15.750 | 0.004 | 20.862 | 0.006 | 37.866 | 0.010 |
| 28 | CMT | 14 | 9.815 | 0.003 | 13.188 | 0.003 | 12.423 | 0.003 | 33.234 | 0.009 | 8.529 | 0.002 | 11.015 | 0.003 | 18.151 | 0.005 | 47.642 | 0.012 | 13.880 | 0.004 | 18.651 | 0.005 | 17.568 | 0.005 | 47.001 | 0.012 |
| 29 | CMT | 15 | 11.236 | 0.003 | 13.665 | 0.003 | 14.414 | 0.004 | 30.660 | 0.008 | 9.568 | 0.002 | 10.010 | 0.003 | 12.595 | 0.003 | 39.463 | 0.010 | 15.890 | 0.004 | 19.325 | 0.005 | 20.385 | 0.005 | 43.359 | 0.011 |
| 30 | Control | 7 | 8.062 | 0.002 | 7.219 | 0.002 | 14.891 | 0.004 | 22.512 | 0.006 | 8.398 | 0.002 | 5.032 | 0.001 | 15.363 | 0.004 | 22.616 | 0.006 | 11.401 | 0.003 | 10.209 | 0.003 | 21.059 | 0.006 | 31.837 | 0.009 |
| 31 | Control | 7 | 9.898 | 0.003 | 12.628 | 0.004 | 13.756 | 0.004 | 22.753 | 0.006 | 7.789 | 0.002 | 9.493 | 0.003 | 10.987 | 0.003 | 18.688 | 0.005 | 13.998 | 0.004 | 17.859 | 0.005 | 19.453 | 0.006 | 32.178 | 0.009 |
| 32 | Control | 7 | 12.498 | 0.004 | 15.338 | 0.004 | 17.835 | 0.005 | 17.882 | 0.005 | 6.788 | 0.002 | 9.318 | 0.003 | 11.893 | 0.003 | 12.932 | 0.004 | 17.674 | 0.005 | 21.691 | 0.006 | 25.222 | 0.007 | 25.289 | 0.007 |
| 33 | Control | 9 | 7.208 | 0.002 | 9.611 | 0.003 | 10.946 | 0.003 | 15.417 | 0.004 | 5.702 | 0.002 | 6.830 | 0.002 | 10.217 | 0.003 | 13.454 | 0.004 | 10.193 | 0.003 | 13.592 | 0.004 | 15.481 | 0.004 | 21.803 | 0.006 |
| 34 | Control | 9 | 7.302 | 0.002 | 10.037 | 0.003 | 12.278 | 0.003 | 19.799 | 0.005 | 6.096 | 0.002 | 7.781 | 0.002 | 11.459 | 0.003 | 18.002 | 0.005 | 10.327 | 0.003 | 14.194 | 0.004 | 17.363 | 0.005 | 28.001 | 0.008 |
| 35 | Control | 9 | 6.876 | 0.002 | 6.922 | 0.002 | 12.870 | 0.003 | 26.155 | 0.007 | 3.683 | 0.001 | 3.828 | 0.001 | 11.752 | 0.003 | 24.679 | 0.006 | 9.724 | 0.003 | 9.789 | 0.003 | 18.200 | 0.005 | 36.989 | 0.010 |
| 36 | Control | 9 | 3.630 | 0.001 | 4.283 | 0.001 | 7.591 | 0.002 | 10.671 | 0.003 | 3.540 | 0.001 | 3.705 | 0.001 | 7.403 | 0.002 | 12.598 | 0.003 | 5.133 | 0.001 | 6.058 | 0.002 | 10.735 | 0.003 | 15.092 | 0.004 |
| 37 | Control | 10 | 6.622 | 0.002 | 8.590 | 0.002 | 11.270 | 0.003 | 15.103 | 0.004 | 4.857 | 0.001 | 6.635 | 0.002 | 12.219 | 0.003 | 18.695 | 0.005 | 9.365 | 0.002 | 12.148 | 0.003 | 15.938 | 0.004 | 21.358 | 0.006 |
| 38 | Control | 11 | 5.628 | 0.001 | 8.151 | 0.002 | 9.658 | 0.003 | 12.968 | 0.003 | 3.788 | 0.001 | 6.390 | 0.002 | 8.389 | 0.002 | 12.842 | 0.003 | 7.960 | 0.002 | 11.527 | 0.003 | 13.659 | 0.004 | 18.340 | 0.005 |
| 39 | Control | 10 | 9.377 | 0.002 | 11.919 | 0.003 | 15.952 | 0.004 | 24.117 | 0.006 | 6.850 | 0.002 | 7.801 | 0.002 | 11.486 | 0.003 | 25.433 | 0.007 | 13.261 | 0.003 | 16.857 | 0.004 | 22.559 | 0.006 | 34.106 | 0.009 |
| 40 | Control | 10 | 10.667 | 0.003 | 10.278 | 0.003 | 21.732 | 0.006 | 27.452 | 0.007 | 7.633 | 0.002 | 6.778 | 0.002 | 21.402 | 0.006 | 34.021 | 0.009 | 15.085 | 0.004 | 14.536 | 0.004 | 30.734 | 0.008 | 38.823 | 0.010 |
| 41 | Control | 9 | 5.414 | 0.001 | 7.629 | 0.002 | 9.656 | 0.003 | 15.384 | 0.004 | 4.792 | 0.001 | 6.391 | 0.002 | 8.368 | 0.002 | 14.799 | 0.004 | 7.656 | 0.002 | 10.789 | 0.003 | 13.655 | 0.004 | 21.757 | 0.006 |
| 42 | Control | 10 | 6.848 | 0.002 | 7.624 | 0.002 | 10.941 | 0.003 | 15.280 | 0.004 | 5.666 | 0.002 | 5.344 | 0.001 | 10.345 | 0.003 | 16.373 | 0.004 | 9.684 | 0.003 | 10.782 | 0.003 | 15.473 | 0.004 | 21.609 | 0.006 |
| 43 | Control | 12 | 5.030 | 0.001 | 7.596 | 0.002 | 11.273 | 0.003 | 21.038 | 0.005 | 4.375 | 0.001 | 5.099 | 0.001 | 8.466 | 0.002 | 19.648 | 0.005 | 7.113 | 0.002 | 10.743 | 0.003 | 15.943 | 0.004 | 29.753 | 0.007 |
| 44 | Control | 12 | 5.883 | 0.002 | 7.400 | 0.002 | 9.145 | 0.002 | 16.071 | 0.004 | 4.085 | 0.001 | 4.808 | 0.001 | 8.841 | 0.002 | 14.592 | 0.004 | 8.320 | 0.002 | 10.465 | 0.003 | 12.932 | 0.003 | 22.728 | 0.006 |
| 45 | Control | 14 | 7.249 | 0.002 | 9.783 | 0.002 | 12.412 | 0.003 | 20.788 | 0.005 | 5.614 | 0.001 | 6.052 | 0.001 | 9.402 | 0.002 | 19.280 | 0.005 | 10.252 | 0.003 | 13.835 | 0.003 | 17.553 | 0.004 | 29.399 | 0.007 |
| 46 | Control | 14 | 5.546 | 0.001 | 4.881 | 0.001 | 9.182 | 0.002 | 21.873 | 0.005 | 2.954 | 0.001 | 2.797 | 0.001 | 8.488 | 0.002 | 18.325 | 0.005 | 7.843 | 0.002 | 6.903 | 0.002 | 12.985 | 0.003 | 30.933 | 0.008 |
| 47 | Control | 14 | 4.770 | 0.001 | 7.759 | 0.002 | 8.824 | 0.002 | 18.074 | 0.004 | 3.067 | 0.001 | 3.984 | 0.001 | 6.850 | 0.002 | 14.932 | 0.004 | 6.745 | 0.002 | 10.973 | 0.003 | 12.478 | 0.003 | 25.560 | 0.006 |
| 48 | Control | 13 | 3.961 | 0.001 | 4.693 | 0.001 | 8.844 | 0.002 | 12.105 | 0.003 | 2.329 | 0.001 | 2.342 | 0.001 | 6.219 | 0.002 | 10.039 | 0.003 | 5.602 | 0.001 | 6.637 | 0.002 | 12.507 | 0.003 | 17.119 | 0.004 |
| 49 | Control | 15 | 3.079 | 0.001 | 3.880 | 0.001 | 8.182 | 0.002 | 19.896 | 0.005 | 3.083 | 0.001 | 3.561 | 0.001 | 7.602 | 0.002 | 19.933 | 0.005 | 4.355 | 0.001 | 5.488 | 0.001 | 11.572 | 0.003 | 28.137 | 0.007 |
| 50 | Control | 16 | 11.565 | 0.003 | 9.171 | 0.002 | 11.041 | 0.003 | 18.670 | 0.005 | 7.824 | 0.002 | 3.672 | 0.001 | 7.872 | 0.002 | 11.295 | 0.003 | 16.356 | 0.004 | 12.970 | 0.003 | 15.615 | 0.004 | 26.403 | 0.007 |
| 51 | Control | 16 | 4.064 | 0.001 | 5.653 | 0.001 | 10.202 | 0.002 | 17.604 | 0.004 | 2.721 | 0.001 | 3.388 | 0.001 | 8.745 | 0.002 | 13.223 | 0.003 | 5.748 | 0.001 | 7.995 | 0.002 | 14.428 | 0.003 | 24.895 | 0.006 |
| 52 | Control | 17 | 7.569 | 0.002 | 7.028 | 0.002 | 10.149 | 0.002 | 18.442 | 0.005 | 5.053 | 0.001 | 3.768 | 0.001 | 9.131 | 0.002 | 22.568 | 0.006 | 10.704 | 0.003 | 9.940 | 0.002 | 14.353 | 0.004 | 26.081 | 0.006 |
| 53 | Control | 17 | 5.111 | 0.001 | 6.720 | 0.002 | 8.258 | 0.002 | 16.067 | 0.004 | 3.476 | 0.001 | 4.081 | 0.001 | 7.402 | 0.002 | 13.754 | 0.003 | 7.228 | 0.002 | 9.503 | 0.002 | 11.678 | 0.003 | 22.723 | 0.006 |

**S1 Table 3. Mean Frequencies of each participant and condition.**

Legend: FT=total frequency; FML= mediolateral frequency; FAP= anteroposterior frequency; OR= open regular condition; CR= closed -regular condition; OF= open-foam condition; CF = closed-foam condition. Empty cels are missing data.

| **Subject** | **GROUP** | **AGE** | **FT OR** | **FT CR** | **FT OF** | **FT CF** | **FML OR** | **FML CR** | **FML OF** | **FML CF** | **FAP OR** | **FAP CR** | **FAP OF** | **FAP CF** |
| --- | --- | --- | --- | --- | --- | --- | --- | --- | --- | --- | --- | --- | --- | --- |
| 1 | CMT | 7 | 0.20 | 0.18 | 0.18 | 0.20 | 0.50 | 0.43 | 0.32 | 0.36 | 0.35 | 0.34 | 0.47 | 0.33 |
| 2 | CMT | 7 | 0.50 | 0.56 | 0.49 | 0.52 | 0.86 | 0.85 | 0.80 | 0.91 | 0.84 | 0.88 | 0.82 | 0.76 |
| 3 | CMT | 7 | 0.49 | 0.39 | 0.54 | 0.38 | 0.80 | 0.76 | 0.87 | 0.70 | 0.73 | 0.72 | 0.89 | 0.93 |
| 4 | CMT | 8 | 0.52 | 0.54 | 0.57 | 0.51 | 0.80 | 0.80 | 0.88 | 0.83 | 0.84 | 0.87 | 0.83 | 0.84 |
| 5 | CMT | 9 | 0.50 | 0.43 | 0.46 | 0.37 | 0.87 | 0.84 | 0.90 | 0.80 | 0.83 | 0.84 | 0.81 | 0.81 |
| 6 | CMT | 9 | 0.61 | 0.77 | 0.60 | 0.69 | 0.83 | 0.89 | 0.73 | 0.81 | 0.87 | 0.89 | 0.70 | 0.84 |
| 7 | CMT | 9 | 0.50 | 0.63 | 0.54 | 0.47 | 0.86 | 0.86 | 0.85 | 0.73 | 0.72 | 0.87 | 0.80 | 0.77 |
| 8 | CMT | 10 | 0.58 | 0.44 | 0.48 | 0.42 | 0.85 | 0.81 | 0.81 | 0.88 | 0.87 | 0.97 | 0.79 | 0.86 |
| 9 | CMT | 10 | 0.38 | 0.44 | 0.41 | 0.40 | 0.84 | 0.74 | 0.89 | 0.76 | 0.78 | 0.89 | 0.78 | 0.87 |
| 10 | CMT | 10 | 0.55 | 0.34 | 0.53 |  | 0.77 | 0.70 | 0.78 |  | 0.70 | 0.78 | 0.83 |  |
| 11 | CMT | 10 | 0.54 | 0.33 | 0.56 | 0.53 | 0.87 | 0.66 | 0.83 | 0.80 | 0.84 | 0.54 | 0.84 | 0.76 |
| 12 | CMT | 10 | 0.20 | 0.20 | 0.38 |  | 0.38 | 0.42 | 0.34 |  | 0.35 | 0.43 | 0.84 |  |
| 13 | CMT | 11 | 0.55 | 0.52 | 0.51 | 0.53 | 0.85 | 0.84 | 0.76 | 0.90 | 0.85 | 0.79 | 0.81 | 0.77 |
| 14 | CMT | 11 | 0.50 | 0.38 | 0.56 | 0.33 | 0.89 | 0.80 | 0.89 | 0.79 | 0.84 | 0.80 | 0.95 | 0.81 |
| 15 | CMT | 12 | 0.53 | 0.71 | 0.51 | 0.62 | 0.88 | 0.84 | 0.88 | 0.80 | 0.81 | 0.80 | 0.81 | 0.78 |
| 16 | CMT | 14 | 0.51 | 0.64 | 0.50 | NaN | 0.80 | 0.79 | 0.83 |  | 0.76 | 0.73 | 0.87 |  |
| 17 | CMT | 14 | 0.43 | 0.52 | 0.39 | 0.52 | 0.87 | 0.79 | 0.87 | 0.80 | 0.81 | 0.79 | 0.87 | 0.79 |
| 18 | CMT | 14 | 0.55 | 0.52 | 0.51 | 0.53 | 0.85 | 0.84 | 0.76 | 0.90 | 0.85 | 0.79 | 0.81 | 0.77 |
| 19 | CMT | 14 | 0.40 | 0.39 | 0.39 | 0.30 | 0.79 | 0.80 | 0.78 | 0.61 | 0.78 | 0.82 | 0.72 | 0.78 |
| 20 | CMT | 15 | 0.56 | 0.51 | 0.55 | 0.52 | 0.76 | 0.83 | 0.85 | 0.83 | 0.79 | 0.81 | 0.77 | 0.77 |
| 21 | CMT | 15 | 0.09 | 0.16 | 0.11 | 0.08 | 0.21 | 0.27 | 0.19 | 0.26 | 0.17 | 0.24 | 0.14 | 0.18 |
| 22 | CMT | 16 | 0.49 | 0.50 | 0.53 | 0.46 | 0.77 | 0.78 | 0.86 | 0.81 | 0.85 | 0.82 | 0.79 | 0.75 |
| 23 | CMT | 16 | 0.13 | 0.15 | 0.26 | 0.27 | 0.30 | 0.41 | 0.61 | 0.56 | 0.21 | 0.22 | 0.35 | 0.43 |
| 24 | CMT | 17 | 0.50 | 0.40 | 0.53 | 0.38 | 0.75 | 0.82 | 0.71 | 0.74 | 0.77 | 0.85 | 0.80 | 0.84 |
| 25 | CMT | 15 | 0.23 | 0.13 | 0.21 | 0.13 | 0.33 | 0.29 | 0.32 | 0.27 | 0.61 | 0.31 | 0.51 | 0.27 |
| 26 | CMT | 11 | 0.33 | 0.58 | 0.59 | 0.15 | 0.37 | 0.67 | 0.63 | 0.37 | 0.43 | 0.65 | 0.71 | 0.31 |
| 27 | CMT | 10 | 0.46 | 0.70 | 0.50 | 0.60 | 0.78 | 0.88 | 0.94 | 0.79 | 0.86 | 0.87 | 0.90 | 0.80 |
| 28 | CMT | 14 | 0.14 | 0.12 | 0.14 | 0.18 | 0.29 | 0.31 | 0.29 | 0.25 | 0.29 | 0.15 | 0.31 | 0.25 |
| 29 | CMT | 15 | 0.15 | 0.11 | 0.20 | 0.22 | 0.28 | 0.23 | 0.34 | 0.34 | 0.29 | 0.23 | 0.35 | 0.43 |
| 30 | Control | 7 | 0.88 | 0.78 | 0.80 | 0.94 | 0.88 | 0.86 | 0.85 | 0.86 | 0.88 | 0.78 | 0.80 | 0.94 |
| 31 | Control | 7 | 0.82 | 0.82 | 0.90 | 0.77 | 0.80 | 0.82 | 0.82 | 0.79 | 0.82 | 0.82 | 0.90 | 0.77 |
| 32 | Control | 7 | 0.65 | 0.87 | 0.52 | 0.76 | 0.42 | 0.83 | 0.26 | 0.83 | 0.65 | 0.87 | 0.52 | 0.76 |
| 33 | Control | 9 | 0.90 | 0.71 | 0.90 | 0.67 | 0.85 | 0.77 | 0.86 | 0.72 | 0.90 | 0.71 | 0.90 | 0.67 |
| 34 | Control | 9 | 0.86 | 0.92 | 0.85 | 0.82 | 0.85 | 0.82 | 0.83 | 0.89 | 0.86 | 0.92 | 0.85 | 0.82 |
| 35 | Control | 9 | 0.85 | 0.84 | 0.81 | 0.85 | 0.72 | 0.74 | 0.86 | 0.84 | 0.85 | 0.84 | 0.81 | 0.85 |
| 36 | Control | 9 | 0.78 | 0.87 | 0.86 | 0.81 | 0.85 | 0.77 | 0.79 | 0.86 | 0.78 | 0.87 | 0.86 | 0.81 |
| 37 | Control | 10 | 0.72 | 0.86 | 0.77 | 0.74 | 0.75 | 0.81 | 0.77 | 0.86 | 0.72 | 0.86 | 0.77 | 0.74 |
| 38 | Control | 11 | 0.81 | 0.88 | 0.87 | 0.88 | 0.86 | 0.85 | 0.88 | 0.82 | 0.81 | 0.88 | 0.87 | 0.88 |
| 39 | Control | 10 | 0.86 | 0.82 | 0.75 | 0.76 | 0.84 | 0.84 | 0.80 | 0.83 | 0.86 | 0.82 | 0.75 | 0.76 |
| 40 | Control | 10 | 0.82 | 0.79 | 0.77 | 0.79 | 0.77 | 0.88 | 0.80 | 0.86 | 0.82 | 0.79 | 0.77 | 0.79 |
| 41 | Control | 9 | 0.78 | 0.76 | 0.85 | 0.81 | 0.85 | 0.81 | 0.91 | 0.81 | 0.78 | 0.76 | 0.85 | 0.81 |
| 42 | Control | 10 | 0.85 | 0.86 | 0.92 | 0.80 | 0.86 | 0.90 | 0.80 | 0.79 | 0.85 | 0.86 | 0.92 | 0.80 |
| 43 | Control | 12 | 0.71 | 0.80 | 0.81 | 0.85 | 0.84 | 0.81 | 0.80 | 0.87 | 0.71 | 0.80 | 0.81 | 0.85 |
| 44 | Control | 12 | 0.95 | 0.87 | 0.85 | 0.72 | 0.85 | 0.89 | 0.81 | 0.71 | 0.95 | 0.87 | 0.85 | 0.72 |
| 45 | Control | 14 | 0.76 | 0.83 | 0.87 | 0.85 | 0.78 | 0.88 | 0.90 | 0.85 | 0.76 | 0.83 | 0.87 | 0.85 |
| 46 | Control | 14 | 0.81 | 0.83 | 0.72 | 0.86 | 0.76 | 0.85 | 0.83 | 0.91 | 0.81 | 0.83 | 0.72 | 0.86 |
| 47 | Control | 14 | 0.82 | 0.91 | 0.88 | 0.85 | 0.73 | 0.75 | 0.89 | 0.79 | 0.82 | 0.91 | 0.88 | 0.85 |
| 48 | Control | 13 | 0.86 | 0.84 | 0.79 | 0.84 | 0.77 | 0.79 | 0.81 | 0.90 | 0.86 | 0.84 | 0.79 | 0.84 |
| 49 | Control | 15 | 0.74 | 0.91 | 0.78 | 0.82 | 0.86 | 0.78 | 0.78 | 0.74 | 0.74 | 0.91 | 0.78 | 0.82 |
| 50 | Control | 16 | 0.88 | 0.92 | 0.85 | 0.79 | 0.91 | 0.83 | 0.83 | 0.77 | 0.88 | 0.92 | 0.85 | 0.79 |
| 51 | Control | 16 | 0.86 | 0.69 | 0.82 | 0.83 | 0.81 | 0.89 | 0.88 | 0.82 | 0.86 | 0.69 | 0.82 | 0.83 |
| 52 | Control | 17 | 0.80 | 0.85 | 0.89 | 0.73 | 0.82 | 0.94 | 0.89 | 0.80 | 0.80 | 0.85 | 0.89 | 0.73 |
| 53 | Control | 17 | 0.82 | 0.77 | 0.87 | 0.76 | 0.87 | 0.86 | 0.83 | 0.78 | 0.82 | 0.77 | 0.87 | 0.76 |

**S1 Table 4. Romberg quotient of each participant and condition.**

Legend: RQ v (CR/OR) = Romberg quotient obtained by open-regular/closed-regular condition; RQ V (CF/OF) = Romberg quotient obtained by open-foam/closed-foam condition. Empty cels are missing data.

| **Subject** | **GROUP** | **AGE** | **RQ v (CR/OR)** | **RQ V (CF/OF)** |
| --- | --- | --- | --- | --- |
| 1 | CMT | 7 | 1.07 | 0.49 |
| 2 | CMT | 7 | 0.75 |  |
| 3 | CMT | 7 | 1.10 | 0.61 |
| 4 | CMT | 8 | 1.27 | 0.61 |
| 5 | CMT | 9 | 1.65 | 0.82 |
| 6 | CMT | 9 | 1.37 | 0.46 |
| 7 | CMT | 9 | 1.51 | 0.53 |
| 8 | CMT | 10 | 2.01 | 0.50 |
| 9 | CMT | 10 | 1.92 | 0.85 |
| 10 | CMT | 10 | 1.93 |  |
| 11 | CMT | 10 | 1.23 | 0.53 |
| 12 | CMT | 10 | 2.07 |  |
| 13 | CMT | 11 | 1.64 | 0.55 |
| 14 | CMT | 11 | 1.54 | 0.49 |
| 15 | CMT | 12 | 1.24 | 0.60 |
| 16 | CMT | 14 | 1.78 |  |
| 17 | CMT | 14 | 0.95 | 0.84 |
| 18 | CMT | 14 | 0.99 |  |
| 19 | CMT | 14 | 1.42 | 0.70 |
| 20 | CMT | 15 | 1.78 | 0.75 |
| 21 | CMT | 15 | 0.98 |  |
| 22 | CMT | 16 | 1.92 | 0.47 |
| 23 | CMT | 16 | 3.22 | 0.39 |
| 24 | CMT | 17 | 1.10 | 0.69 |
| 25 | CMT | 15 | 1.17 | 0.95 |
| 26 | CMT | 11 | 1.76 | 0.51 |
| 27 | CMT | 10 | 0.79 | 0.55 |
| 28 | CMT | 14 | 1.34 | 0.37 |
| 29 | CMT | 15 | 1.22 | 0.47 |
| 30 | Control | 7 | 0.90 | 0.66 |
| 31 | Control | 7 | 1.28 | 0.60 |
| 32 | Control | 7 | 1.23 | 1.00 |
| 33 | Control | 9 | 1.33 | 0.71 |
| 34 | Control | 9 | 1.37 | 0.62 |
| 35 | Control | 9 | 1.01 | 0.49 |
| 36 | Control | 9 | 1.18 | 0.71 |
| 37 | Control | 10 | 1.30 | 0.75 |
| 38 | Control | 11 | 1.45 | 0.74 |
| 39 | Control | 10 | 1.27 | 0.66 |
| 40 | Control | 10 | 0.96 | 0.79 |
| 41 | Control | 9 | 1.41 | 0.63 |
| 42 | Control | 10 | 1.11 | 0.72 |
| 43 | Control | 12 | 1.51 | 0.54 |
| 44 | Control | 12 | 1.26 | 0.57 |
| 45 | Control | 14 | 1.35 | 0.60 |
| 46 | Control | 14 | 0.88 | 0.42 |
| 47 | Control | 14 | 1.63 | 0.49 |
| 48 | Control | 13 | 1.18 | 0.73 |
| 49 | Control | 15 | 1.26 | 0.41 |
| 50 | Control | 16 | 0.79 | 0.59 |
| 51 | Control | 16 | 1.39 | 0.58 |
| 52 | Control | 17 | 0.93 | 0.55 |
| 53 | Control | 17 | 1.31 | 0.51 |
